# Associations of parental academic achievement pressure, support, and school climate with hikikomori tendency among high school students

**DOI:** 10.3389/fpsyg.2026.1745661

**Published:** 2026-03-11

**Authors:** Selda Meydan, Fatih Cebeci, Aylin Arıcı, Sinem Arslankoç, Şeyma Karakaya Altıok, Sayra Lotfi, Taner Artan, Gökçehan Gelener, Nazlıcan Kırcı

**Affiliations:** 1 Department of Social Work, School of Health Sciences, Istanbul Medipol University, Istanbul, Türkiye; 2 Department of Social Work, Faculty of Health Sciences, Istanbul Medipol University, Istanbul, Türkiye; 3 UNEC Social Work and Social Innovations Research Center, Azerbaijan State University of Economics, Baku, Azerbaijan; 4 Department of Social Work, Faculty of Health Sciences, Istanbul University-Cerrahpasa, Istanbul, Türkiye

**Keywords:** hikikomori, parental academic achievement pressure, parental academic achievement support, school climate, social withdrawal

## Abstract

**Objective:**

The purpose of this study is to examine the associations between parental academic achievement pressure, parental academic achievement support, school climate, and hikikomori tendency among high school students.

**Methods:**

Employing a quantitative method with a predictive correlational design, the study was conducted with a sample of 404 high school students in Istanbul during the 2023–2024 academic year. Data were collected using the Socio-Demographic Information Form, the 25-item Hikikomori Questionnaire (HQ-25), the Parents’ Academic Achievement Pressure and Support Scale, and the School Climate Questionnaire-High School Form. Correlation analyses and hierarchical multiple regression analyses were performed to examine the relationships among the study variables.

**Results:**

Correlation analyses indicated a significant positive association between hikikomori tendency and parental academic achievement pressure (*r* = 0.237, *p* < 0.01), as well as significant negative associations with parental academic achievement support (*r* = −0.345, *p* < 0.01) and school climate (*r* = −0.262, *p* < 0.01). Multiple regression analyses showed that parental academic achievement support and school climate were negatively associated with hikikomori tendency, whereas parental academic achievement pressure was positively associated. The model accounted for approximately 19% of the variance in students’ hikikomori tendency (*R*^2^ = 0.189). In addition, students who perceived themselves as academically unsuccessful, had repeated a grade, or reported low socioeconomic status exhibited significantly higher levels of hikikomori tendency (*p* < 0.05).

**Conclusion:**

The findings indicate that family- and school-related contextual factors are meaningfully associated with adolescents’ social withdrawal tendencies. While parental academic achievement pressure is linked to higher levels of hikikomori tendency, parental academic achievement support and a positive school climate are associated with lower levels of social withdrawal. Given the cross-sectional design of the study, these findings should be interpreted as correlational rather than causal. The results provide empirical support for the development of preventive and supportive practices within educational and mental health contexts.

## 1 Introduction

In today’s educational systems, the increasingly competitive structure and exam-oriented assessment approaches exert intense academic pressure on students, significantly amplifying parental expectations regarding their children’s academic success (Tatlı and Atmaca, 2023). Particularly, the necessity of progressing through educational stages via centralized examinations increases parental academic pressure at earlier ages, which in turn adversely affects students’ psychosocial development (Cheung and Pomerantz, 2011; Owens, 2021). In family environments where parental pressure is intense, students often feel that they are evaluated solely based on their achievements; this perception can lead to negative psychological outcomes such as anxiety, burnout, and social withdrawal.

In contrast, supportive parenting styles and a healthy school climate have been shown to enhance students’ levels of motivation, sense of belonging, and psychological resilience (Concina et al., 2024; Murayama et al., 2016; Daily et al., 2019). However, when a negative school climate, weak peer relationships, and high academic pressure converge, behaviors of social isolation and introversion become more prevalent among individuals. Within this context, *hikikomori* emerges as a significant issue characterized by prolonged social withdrawal and home confinement. It is not only an individual phenomenon but also one that is closely associated with environmental factors such as parental attitudes and school climate (Bonnaire and Roignot, 2023; Kato, 2020).

### 1.1 Parental academic achievement pressure and support

Academic pressure is defined as the imposition of achievement standards set by parents onto their children, encompassing efforts to compel students to attain higher levels of performance (Gülbetekin and Tunç, 2022; Kapıkıran, 2016). Moreover, academic pressure refers to the stress and discomfort arising from school, family, and society-based expectations throughout the learning process (Jiang et al., 2022). This pressure may manifest through excessive parental expectations for hard work, comparisons with others, and the devaluation of a child’s accomplishments (Kapıkıran, 2020). Additionally, academic achievement pressure may result from a mismatch between an individual’s competencies and external expectations (Zhao et al., 2023). Therefore, academic failure should not be attributed solely to the student; it must also be evaluated in the context of parental and environmental pressures.

It has been suggested that academic achievement pressure adversely affects both students’ performance levels and their psychological wellbeing (Tatlı and Atmaca, 2023). Such pressure has been shown to lead to anxiety, burnout, and lower life satisfaction, whereas supportive parental attitudes are associated with increased academic resilience and motivation (Daily et al., 2019; Murayama et al., 2016). Parental academic support, in particular, plays a critical role in shaping students’ motivation, academic performance, and adjustment processes (Jang and Suh, 2021). This form of support may take various forms, such as involvement in school activities, supervision of homework, and the provision of a conducive study environment (Morkoyunlu and Konyalıoğlu, 2020), generally facilitating students’ active engagement in the academic process.

However, overly intrusive or excessive forms of support can hinder the development of students’ independence, increase their levels of anxiety, and negatively impact their psychological adjustment (Flouri, 2006; Jang and Suh, 2021; Pomerantz and Wang, 2009; Sy et al., 2013).

### 1.2 School climate

In addition to parental academic achievement pressure and support, one of the key environmental factors influencing children’s educational experiences is school climate. Research defines school climate as a multidimensional construct encompassing the social, emotional, and physical conditions that support learning (Bottiani et al., 2020; Haynes et al., 1997). This construct includes elements such as student-teacher relationships, peer interactions, learning environment, parental pressure and support, as well as the values and norms of the school. Collectively, these components constitute an ecological context that shapes individuals’ cognitive, social, and psychological development (Vaid et al., 2023).

A positive school climate has been significantly associated with favorable educational outcomes such as adaptive motivation, school engagement, and academic achievement (Korpershoek et al., 2020). Indeed, numerous studies have demonstrated that healthy school climate domains are strongly correlated with academic success (Bahçetepe and Meşeci Giorgetti, 2015; Bhat and Mir, 2018; Daily et al., 2019; Ruiz et al., 2018).

### 1.3 The relationship between hikikomori tendency and parental academic pressure, parental academic support, and school climate

The relevant literature indicates that parental academic achievement pressure, parental support, and school climate exert significant influences across various domains of students’ lives. Although these effects can at times be positive, they have also been associated with adverse outcomes such as academic failure, anxiety, depression, disruption in social relationships, and loneliness. Hikikomori was first systematically conceptualized in Japan and gained wider academic recognition through Saito’s seminal work, which described the phenomenon as prolonged disengagement from education or employment accompanied by persistent home-based isolation lasting at least six months (Saito, 1998). Subsequent research has further elaborated this definition by emphasizing core behavioral characteristics such as sustained withdrawal from social participation, avoidance of interpersonal roles, and long-term confinement within the home environment (Tamaki, 2013; Teo et al., 2018). Although certain symptomatic features of hikikomori may overlap with depressive conditions, including reduced motivation and diminished social activity, the literature consistently indicates that hikikomori represents a distinct psychosocial pattern with unique developmental and contextual dynamics. Empirical studies further demonstrate that individuals exhibiting hikikomori-related behaviors frequently experience elevated psychological distress, marked functional impairment, and deterioration in overall wellbeing (Kato et al., 2011; Teo et al., 2015). From this perspective, parent–child relationships, parental expectations, and failures experienced in school life can trigger the phenomenon of *hikikomori*, a form of social isolation closely associated with loneliness (Coşan, 2021).

Initially identified in Japan in the early 2000s, hikikomori was originally considered a culture bound phenomenon. However, over time, it has been observed in various societies and has come to be recognized as a global public health concern (Tamaki, 2013; Teo et al., 2018). In recent years, hikikomori has gained attention in Türkiye as an education related social phenomenon. In societies where education is perceived as a highly competitive process and failure often leads to social exclusion, individuals are reported to exhibit tendencies toward withdrawal and hikikomori like behaviors (Furlong, 2008; Saito, 2013).

Building on this conceptual background, the present study aims to empirically examine whether parental academic achievement pressure, parental academic achievement support, and school climate serve as significant predictors of hikikomori tendency among high school students. The literature suggests that excessive academic pressure and controlling forms of parental support characterized by high performance expectations may increase students’ anxiety, thereby fostering fear of failure and a tendency toward social withdrawal (Furlong and Morrison, 2000; Teo, 2010). Furthermore, factors such as a negative school climate, insufficient teacher support, and peer bullying have been found to reinforce this process (Kato et al., 2012). In contrast, supportive parental attitudes and a positive school climate enhance students’ academic and social competencies, thereby serving a protective function against the development of hikikomori (Uchida and Norasakkunkit, 2015).

### 1.4 Theoretical framework: ecological systems theory

Bronfenbrenner’s Ecological Systems Theory, which explains human development within the framework of dynamic interactions between individuals and their environments, constitutes the theoretical foundation of this study. The ecological approach asserts that individual behavior is shaped through the reciprocal interaction of internal and environmental factors and conceptualizes the individual as a multidimensional psychosocial being (Orman et al., 2022). Within this framework, adolescents’ psychosocial outcomes should be understood in relation to their immediate environments, particularly family and school contexts. From an ecological perspective, parental academic achievement pressure and parental academic achievement support represent fundamental microsystem processes that influence adolescents’ emotional adjustment and social functioning (Kapıkıran, 2016; Cebeci, 2023). Similarly, school climate reflects the relational, social, and psychological quality of the school environment and plays a decisive role in fostering students’ sense of belonging and participation (Thapa et al., 2013). A positive school climate not only enhances academic performance but also strengthens school attachment, thereby serving a protective function against risk factors such as absenteeism and school dropout (Cebeci, 2023; Wang and Degol, 2016).

Ecological Systems Theory also emphasizes the interaction between multiple environments. In this regard, the combined influence of family expectations, parenting attitudes, and school experiences may either increase or decrease adolescents’ psychosocial adjustment and vulnerability to social withdrawal. Accordingly, hikikomori tendency should be understood not merely as an individual characteristic but also as a response shaped by environmental conditions. Research highlights that adolescents’ social adjustment is influenced less by isolated environmental factors than by the interactions between microsystems such as family and school (mesosystem) (Poole, 2025). This multilayered structure directly aligns with Bronfenbrenner’s ecological perspective in explaining social withdrawal behaviors (Yu et al., 2024). Within this ecological framework, the present study focuses on the relationships among family processes, school climate, and hikikomori tendency.

### 1.5 The present study

A review of the literature reveals that the concepts of hikikomori, parental academic achievement pressure and support, and school climate are typically addressed independently from one another. Although various studies have examined the relationships between each of these variables and different psychosocial outcomes, there is a notable scarcity both nationally and internationally of research adopting a holistic approach that evaluates all four factors in conjunction.

At the international level, studies such as those by Wang and Holcombe (2010), Kaçmaz et al. (2025), and Neoh et al. (2023) have explored the relationships between hikikomori and a range of individual and environmental factors. However, these studies have not examined the combined effects of these specific variables. In the Turkish context, there has been a growing body of research on hikikomori since 2017, particularly in the fields of health sciences, social sciences, and digital culture (Coşan, 2021; Demir, 2017; Güzel-Gürbüz et al., 2022; Kasak et al., 2022; Kulaberoğlu and Hocaoğlu, 2024; Onaral, 2020). However, these studies have not yet considered the integrative impact of key social environmental variables on hikikomori tendencies. These variables include parental academic achievement pressure, parental academic support, and school climate.

High school students constitute a relevant group for examining hikikomori tendency, as academic demands, performance evaluation, and parental academic expectations become particularly salient at this educational stage. Within this context, school climate and family related academic processes play a central role in students’ daily functioning. Therefore, the present study examines hikikomori tendency within a school-based academic framework. While the participants are in the adolescent age range, the study does not adopt a developmental focus and instead situates social withdrawal tendencies within the institutional context of high school education.

**Figure 1:**
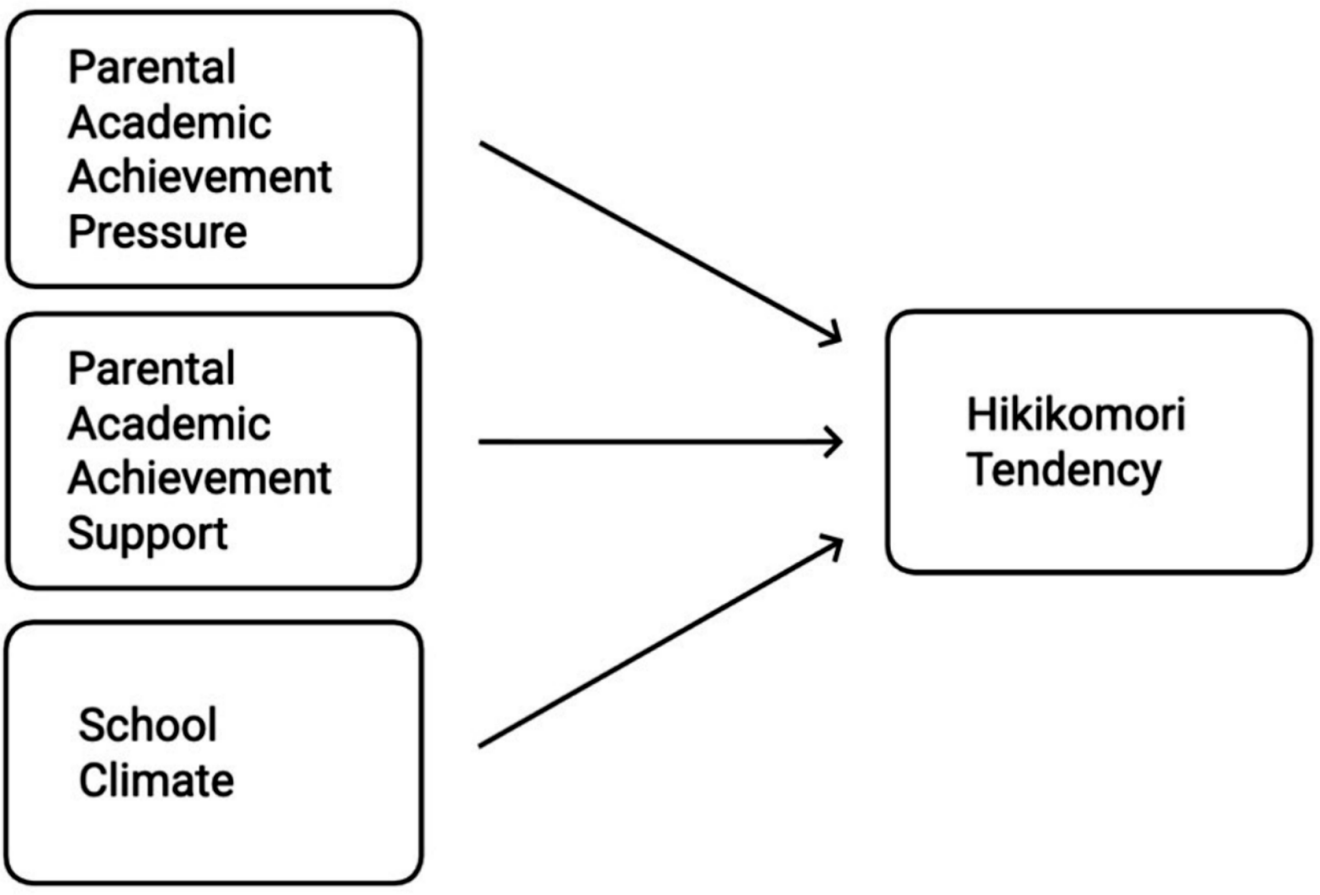
Conceptual model illustrating the predictive roles of parental academic achievement pressure, parental academic achievement support, and school climate on hikikomori tendency.

The aim of the present study is to examine the associations between parental academic achievement pressure, parental academic achievement support, school climate, and hikikomori tendency among high school students. Adopting a multidimensional perspective, the study analyzes the predictive effects of these school and family-based variables on social withdrawal behaviors. Furthermore, this study aims to offer an original and holistic framework for understanding the concept of hikikomori within the Turkish context. Through this approach, it seeks to address a significant gap in the literature and contribute to the development of preventive and intervention-based strategies for policymakers, educators, and mental health professionals at the national level. In this regard, the present study represents one of the pioneering efforts to investigate, within the Turkish context, the relationships among parental academic achievement pressure, parental academic achievement support, school climate, and hikikomori tendency in a comprehensive framework. By demonstrating that hikikomori is a form of social isolation that transcends cultural boundaries, particularly in societies where academic stress is pervasive, this study contributes to filling theoretical gaps in the literature. Moreover, it offers insights that can inform preventive interventions in the domains of educational policy and mental health practices. The conceptual model proposed in the study is illustrated in Figure 1.

## 2 Materials and methods

### 2.1 Research model

This study was designed to examine the relationships among parental academic achievement pressure, parental academic achievement support, and school climate as factors predicting hikikomori tendency in high school students. A quantitative research method was employed as the research approach. The quantitative method is concerned with issues related to research design, measurement, and sampling, and it involves a deductive approach that emphasizes the detailed planning of data collection and analysis procedures prior to implementation (Neuman, 2014). As the research design, a predictive correlational design was adopted. This design not only investigates the relationships among variables but also aims to test the extent to which certain variables can predict the tendency toward hikikomori.

### 2.2 Participants

The study population consisted of high school students enrolled in schools located in Istanbul during the 2023–2024 academic year. According to data from the Ministry of National Education, the number of high school students in Istanbul during this period was 1,217,806 (MEB, 2024). To obtain a sample with a high level of representativeness, a probability-based sampling method simple random sampling was employed. This method ensures that each potential participant has an equal probability of being selected, thereby increasing the likelihood that the sample accurately represents the general population (Büyüköztürk et al., 2008). Within the framework of this sampling method, the names of high schools constituting the research population were entered into a computer program that allows for random selection. Subsequently, students from the randomly selected schools were invited to participate in the study, thereby forming the research sample. Care was taken to ensure representation across different types of high schools. As a result, the sample size was determined to be both representative of the population and sufficient in terms of statistical power.

Approximately 800 participants were initially surveyed in this study. However, due to data quality issues frequently observed in adolescent samples such as missing responses, random answering, and marking the same option across all items a detailed data screening and cleaning procedure was conducted. During this process, forms containing substantial missing data, responses considered inconsistent, or cases deemed unsuitable for analysis were excluded. As a result, all analyses were performed using data from 404 participants. This procedure was implemented to enhance the validity and reliability of the analyses (Table 1).

**Table 1:** Participant characteristics.

Variable category		*f*	%
Gender	Female	181	44.8
	Male	223	55.2
Age	11–15 years	199	49.3
	16–19 years	205	50.7
Grade level	Preparatory	2	0.5
	9th grade	135	33.4
	10th grade	120	29.7
	11th grade	76	18.8
	12th grade	71	17.6
School type	Anatolian high school	298	73.8
	Anatolian imam hatip high school	28	6.9
	Vocational and technical Anatolian high school	78	19.3
Number of siblings	Only child	37	9.2
	Two siblings	181	44.8
	Three siblings	125	30.9
	Four siblings	44	10.9
	Five or more siblings	17	4.2
Self-Perceived Academic Success	Agree	244	60.4
	Neutral	133	32.9
	Disagree	27	6.7
Family’s economic status	Low	24	5.9
	Middle	347	85.9
	High	33	8.2
Parental Vital Status	Both alive	390	96.5
	Only mother alive	12	3.0
	Only father alive	2	0.5
Parental Co-residence	Yes	351	86.9
	No	53	13.1
Living Arrangement	With mother	36	8.9
	With father	16	4.0
	With both parents	335	82.9
	Other	17	4.2
Choosing School Willingly	Yes	309	76.5
	No	95	23.5
Grade Repetition	Yes	27	6.7
	No	377	93.3
Absenteeism (without valid excuse)	0 day	177	43.8
	1–5 days	178	44.1
	6–10 days	30	7.4
	11–15 days	7	1.7
	16 days or more	12	3.0
Mother’s Educational Level	Illiterate	9	2.3
	Literate (without formal schooling)	10	2.5
	Primary school	80	20.3
	Secondary school	59	15.0
	High school	124	31.5
	University and above	112	28.4
Mother’s Employment Status	Employed	166	42.1
	Unemployed	228	57.9
Father’s Educational Level	Illiterate	5	1.3
	Literate (without formal schooling)	8	2.1
	Primary school	55	14.2
	Secondary school	74	19.2
	High school	119	30.8
	University and above	125	32.4
Father’s Employment Status	Employed	329	85.2
	Unemployed	57	14.8

### 2.3 Data collection tools

#### 2.3.1 Socio-demographic information form

This form was developed by the researchers based on a review of the relevant literature. It consists of a total of 17 items that gather information about participants’ age, gender, grade level, number of siblings, self-perceived academic achievement, and various personal details related to their parents.

#### 2.3.2 The 25-item hikikomori questionnaire (HQ-25)

This scale was developed by Teo et al. (2018) to assess hikikomori as a form of severe social withdrawal. The Turkish adaptation and the validity-reliability study of the scale were conducted by Gündoğmuş et al. (2021). Comprising a total of 25 items, the scale is based on a 5-point Likert format (0 = Strongly Disagree, 4 = Strongly Agree) and includes three subdimensions: socialization, isolation, and emotional support. Items 4, 7, 10, 15, 21, and 25 are reverse coded. Each item is scored on a scale from 0 to 4, resulting in a total possible score ranging from 0 to 100. Regarding the subdimensions, the socialization subscale includes 11 items, the isolation subscale contains 8 items, and the emotional support subscale comprises 6 items. The internal consistency of the original scale was reported with a Cronbach’s alpha coefficient of 0.96. In the Turkish adaptation study by Gündoğmuş et al. (2021), the coefficient was calculated as 0.91. These findings indicate that the scale is a reliable and valid measurement tool.

#### 2.3.3 Parents’ academic achievement pressure and support scale (PAAPSS)

This scale was developed by Kapıkıran (2016) to measure the level of pressure and support parents exert on their children to achieve the academic success they themselves desire. The development of the scale is based on items concerning academic pressure and support from a preliminary study conducted by Kapıkıran and Kapıkıran (2009). In the current version of the scale, some items from the previous version were revised and additional items were incorporated. Accordingly, the Parents’ Academic Achievement Pressure and Support Scale (PAAPSS) was structured into two subscales: Parental Academic Achievement Pressure (PAAP) and Parental Academic Achievement Support (PAAS). As expected, the exploratory factor analysis (EFA) revealed a two-factor structure consisting of pressure and support dimensions. The first factor (pressure) includes 10 items, while the second factor (support) includes 5 items, making a total of 15 items. The scale uses a 5-point Likert-type format (1 = Never, 2 = Rarely, 3 = Sometimes, 4 = Often, 5 = Always). The overall Cronbach’s alpha reliability coefficient for the scale was calculated as 0.82. Additionally, the Cronbach’s alpha for the Parental Academic Achievement Pressure subscale was 0.84, and for the Parental Academic Achievement Support subscale it was 0.71.

#### 2.3.4 School climate questionnaire high school form

This scale was developed by Haynes et al. (1997) at the Yale Child Study Center to assess general school adjustment and the quality of relationships between students and adults within the school environment. The Turkish adaptation and the validity-reliability study of the scale was conducted by Bugay et al. (2015). The scale consists of 42 items and uses a 5-point Likert format (1 = Strongly Agree, 5 = Strongly Disagree). It comprises six subscales: use of resources, order and discipline, parental involvement, school building, peer relationships, and student-teacher relationships. Items 1, 3, 7, 14, 18, 20, 25, 31, 32, 34, 35, 39, 40, and 41 are reverse coded, as they reflect negative statements regarding school climate. Each item can receive a score ranging from 1 to 5, with higher average scores indicating more positive perceptions. For instance, an average score of around 4.5 on the student-teacher relationship subscale would suggest high levels of trust, respect, and perceived value between students and teachers. The internal consistency of the scale was calculated with a Cronbach’s alpha coefficient of 0.91, indicating high reliability.

### 2.4 Data collection

Prior to the data collection process, the necessary ethical approval for this study was granted by the Scientific Research Ethics Committee for Social Sciences at Istanbul Medipol University on April 22, 2024 (Decision No: 47). Following this, research permission was secured from the Istanbul Provincial Directorate of National Education on May 27, 2024, as the study involved high school students. Upon completion of the approval procedures, the high schools where data collection would take place were identified, and preliminary meetings were conducted with school administrations. Based on these meetings, the questionnaire forms were distributed to the students, and the data collection process was initiated. The data were collected in face-to-face settings, with each session taking approximately 15–20 min per participant.

Researchers conducting studies in the field of social sciences are expected to adhere to a range of ethical standards, including ensuring voluntary participation, obtaining informed consent, protecting confidentiality and anonymity, and avoiding any harm to participants as a result of their involvement in the research. In accordance with these ethical principles, this study was conducted on the basis of informed consent and voluntary participation. Moreover, since all participants were under the age of 18, parental or guardian consent was also obtained. Data were collected between October 21, 2024, and October 31, 2024.

### 2.5 Data analysis

Before proceeding to the main analyses, descriptive statistics for the study variables were examined (see Table 2). The variable means ranged from 2.44 to 3.84, skewness values ranged from −0.77 to 0.49, and kurtosis values ranged from −0.24 to 0.62. Since both skewness and kurtosis coefficients fall within the acceptable range of −1 to +1, the distributions of the variables were considered to be approximately normal (Hair et al., 2013). Therefore, parametric statistical methods were used in the analysis. To compare the hikikomori, school climate, parental academic achievement pressure, and parental academic achievement support scores across socio-demographic variables, independent samples *t*-tests and one-way ANOVA were conducted. For variables showing significant differences in the one-way ANOVA, Scheffé *post-hoc* tests were applied to identify the source of the differences. Additionally, the study examined the predictive roles of parental academic achievement pressure, parental academic achievement support, and school climate on hikikomori tendency. To control for potential confounding effects, a hierarchical multiple regression analysis was conducted. In the first step, school type and family economic status were entered as control variables. In the second step, the main study variables were entered into the model. Categorical control variables were dummy coded prior to the analysis. Prior to the analysis, the fundamental assumptions of regression were assessed. The Durbin-Watson statistic was found to be 1.836, VIF values ranged from 1.087 to 1.123, and Tolerance values ranged from 0.890 to 0.920. These coefficients indicated that the assumptions of no autocorrelation and no multicollinearity were met (Hair et al., 2013). Therefore, the analysis and interpretation of the data were continued accordingly. All statistical analyses were conducted using SPSS version 22, and the significance level was set at 0.05.

**Table 2:** Descriptive statistics.

Variable	*n*	Mean	SD	Skewness	Kurtosis
PAAPS	404	2.49	0.92	0.49	−0.24
PAASS	404	3.84	1.01	−0.77	0.04
Hikikomori	404	2.44	0.60	0.47	0.62
School Climate	404	2.89	0.52	−0.02	0.56

The values reported for the hikikomori variable in Table 2 represent the item-level mean of the HQ-25 scale. As the scale consists of 25 items, the reported item mean of 2.44 corresponds to an approximate total score of 61 (2.44 × 25 = 61). Because clinical cutoff scores are defined based on total scale scores, HQ-25 total scores derived from the sum of the 25 items were also calculated to enhance interpretability. The recommended cutoff score of 42 for possible clinical-level hikikomori (Teo et al., 2018) was therefore used as a reference point. However, in the present study, the findings are interpreted as indicators of social withdrawal tendencies and risk levels rather than as diagnostic evidence.

## 3 Results

Table 3 presents the results of independent samples *t-*tests and one-way analysis of variance (ANOVA) conducted to compare students’ scores on hikikomori tendency, perceptions of school climate, parental academic achievement pressure, and parental academic achievement support across socio-demographic variables. A statistically significant difference was found in school climate scores by grade level (*p* < 0.01). According to the *post-hoc* Scheffé test, 11th and 12th grade students reported significantly higher school climate scores compared to 9th grade students, with a small to moderate magnitude of effect (*η*^2^ = 0.02). Analyses by school type revealed significant differences in hikikomori tendency, parental pressure, and parental support scores (*p* < 0.05). In particular, students attending Anatolian High Schools and Anatolian Imam Hatip High Schools reported higher parental support scores than those attending Vocational and Technical High Schools, reflecting a small to moderate practical effect (*η*^2^ = 0.03).

**Table 3:** Comparison of research variables according to socio-demographic variables.

Variable	Group	*f*	Hikikomori				School climate				PAAPS				PAASS			
			M	SD	t/F	*p*	M	SD	t/F	*p*	M	SD	t/F	*p*	M	SD	t/F	*p*
Gender	Female	181	2.42	0.59	−0.495	0.621	2.88	0.52	−0.465	0.402	2.30	0.93	-3.727	**0.000**	3.88	1.02	0.737	0.461
	Male	223	2.45	0.62			2.90	0.51			2.64	0.88			3.81	1.00		
Age	11–15 years	199	2.49	0.58	2.400	0.122	2.94	0.51	2.855	0.092	2.49	0.86	0.001	0.997	3.85	1.01	0.052	0.819
	16–19 years	205	2.39	0.62			2.85	0.52			2.49	0.97			3.83	1.01		
Grade level	Preparatory *	2	2.00	0.62	1.221	0.302	3.49	0.82	5.480	**0.001**	1.40	0.28	2.434	0.065	4.50	0.71	0.237	0.870
	9	135	2.47	0.59			3.00	0.48			2.51	0.88			3.80	1.01		
	10	120	2.39	0.58			2.92	0.54			2.46	0.88			3.88	1.05		
	11	76	2.53	0.68			2.77	0.52			2.70	1.02			3.79	1.02		
	12	71	2.39	0.58			2.75	0.46			2.30	0.91			3.89	0.97		
School Type	Anatolian school	298	2.39	0.60	3.710	**0.025**	2.89	0.53	0.670	0.512	2.42	0.95	3.559	**0.029**	3.93	0.97	7.162	**0.001**
	Anatolian I. H. school	28	2.60	0.69			2.80	0.58			2.78	0.83			3.91	1.01		
	Vocational and technical A. school	78	2.57	0.56			2.93	0.42			2.66	0.76			3.45	1.09		
Number of siblings	Only child	37	2.35	0.62	1.497	0.202	2.97	0.67	0.460	0.765	2.51	1.07	1.988	0.096	3.81	1.12	2.274	0.061
	Two siblings	181	2.39	0.61			2.91	0.54			2.38	0.87			4.00	0.91		
	Three siblings	125	2.49	0.54			2.86	0.44			2.51	0.92			3.71	1.05		
	Four siblings	44	2.51	0.71			2.85	0.43			2.75	0.88			3.69	1.06		
	Five or more siblings	17	2.67	0.57			2.89	0.61			2.79	0.98			3.54	1.14		
Self-perceived academic success	Agree	244	2.33	0.57	16.433	**0.000**	2.93	0.52	3.312	**0.037**	2.39	0.90	4.820	**0.009**	4.08	0.92	21.710	**0.000**
	Neutral	133	2.55	0.58			2.86	0.51			2.59	0.90			3.57	1.02		
	Disagree	27	2.92	0.70			2.67	0.43			2.88	1.03			3.04	0.99		
Family’s economic status	Low	24	2.87	0.58	7.254	**0.001**	2.79	0.47	1.314	0.270	2.54	0.87	0.236	0.790	3.40	1.21	2.446	0.088
	Middle	347	2.42	0.58			2.89	0.51			2.48	0.91			3.87	0.98		
	High	33	2.32	0.76			3.01	0.56			2.58	1.06			3.85	1.10		
Parental vital status	Both alive	390	2.44	0.61	0.361	0.718	2.89	0.52	−0.186	0.852	2.48	0.91	-1.548	0.122	3.85	1.00	1.060	0.290
	Only mother alive	12	2.38	0.59			2.92	0.55			2.89	1.02			3.53	1.24		
	Only father alive*	2	2.56	0.11			2.89	0.22			2.80	0.14			4.30	0.71		
Parental co-residence	Yes	351	2.45	0.60	0.501	0.617	2.91	0.52	1.750	0.081	2.48	0.92	−0.474	0.636	3.86	1.01	0.780	0.436
	No	53	2.40	0.63			2.78	0.50			2.55	0.91			3.74	1.05		
Living arrangement	With mother	36	2.39	0.69	1.150	0.329	2.93	0.50	1.546	0.202	2.53	0.91	0.067	0.977	3.49	1.22	2.310	0.076
	With father	16	2.66	0.77			2.67	0.55			2.56	0.73			3.53	1.23		
	Mother and father	335	2.44	0.59			2.89	0.52			2.48	0.92			3.89	0.97		
	Other	17	2.29	0.59			3.04	0.41			2.53	1.09			3.95	0.94		
Choosing school willingly	Yes	309	2.42	0.58	-1.278	0.202	2.95	0.50	4.485	**0.000**	2.37	0.85	−4.670	**0.000**	3.96	0.96	4.275	**0.000**
	No	95	2.51	0.67			2.69	0.51			2.86	1.04			3.46	1.07		
Grade repetition	Yes	27	2.88	0.80	4.026	**0.000**	2.72	0.37	−1.793	0.074	2.77	0.99	1.651	0.100	2.92	1.09	−5.056	**0.000**
	No	377	2.41	0.58			2.90	0.52			2.47	0.91			3.91	0.97		
Absenteeism	0 day	177	2.36	0.57	2.036	0.108	2.95	0.50	1.282	0.280	2.53	0.98	0.501	0.682	3.97	0.90	2.294	0.077
	1–5 days	178	2.51	0.58			2.85	0.55			2.43	0.87			3.77	1.06		
	6–10 days	30	2.49	0.84			2.88	0.41			2.54	0.85			3.54	1.13		
	11 days or more	19	2.49	0.56			2.81	0.48			2.60	0.91			3.74	1.19		
Mother’s educational level	Illiterate	9	2.78	0.47	2.240	0.050	2.78	0.44	2.786	**0.017**	2.69	0.72	2.194	0.054	3.58	1.21	2.409	**0.036**
	Literate (without formal schooling)	10	2.63	0.57			2.80	0.45			2.62	0.67			3.52	0.80		
	Primary school	80	2.53	0.60			2.95	0.53			2.43	0.86			3.62	1.11		
	Secondary school	59	2.35	0.65			3.05	0.44			2.34	0.78			3.80	1.03		
	High school	124	2.47	0.54			2.78	0.48			2.69	1.00			3.85	1.01		
	University and above	112	2.33	0.65			2.91	0.56			2.35	0.95			4.08	0.91		
Mother’s employment status	Employed	166	2.34	0.56	−2.787	**0.006**	2.85	0.52	−1.311	0.191	2.41	0.92	−1.394	0.164	3.95	0.94	1.597	0.111
	Unemployed	228	2.51	0.63			2.92	0.52			2.54	0.92			3.78	1.06		
Father’s educational level	Illiterate	5	2.98	0.89	1.975	0.082	2.61	0.70	0.634	0.674	2.86	1.32	0.849	0.516	3.28	1.66	3.305	**0.006**
	Literate (without formal schooling)	8	2.46	0.38			2.83	0.41			2.85	0.82			3.98	0.93		
	Primary school	55	2.56	0.56			2.85	0.49			2.45	0.98			3.67	1.13		
	Secondary school	74	2.48	0.58			2.95	0.51			2.42	0.78			3.61	1.03		
	High school	119	2.43	0.57			2.92	0.49			2.57	0.91			3.86	0.99		
	University and above	125	2.34	0.66			2.87	0.57			2.42	0.97			4.11	0.88		
Father’s employment status	Employed	329	2.42	0.62	−1.351	0.177	2.90	0.53	0.169	.866	2.50	0.93	0.472	0.637	3.89	1.00	1.309	0.191
	Unemployed	57	2.54	0.50			2.88	0.49			2.43	0.86			3.70	1.06		

*Excluded from comparison due to small sample size.

Bold values indicate statistically significant results (*p* < 0.05 and *p* < 0.01).

In terms of self-perceived academic achievement, statistically significant differences were found across all four dependent variables (*p* < 0.05). Students who perceived themselves as academically successful reported lower hikikomori scores, higher school climate and parental support scores, and lower parental pressure scores. The effect of self-perceived academic success on hikikomori tendency was of moderate magnitude (*η*^2^ = 0.08), indicating meaningful differences between groups. Conversely, those who perceived themselves as academically unsuccessful exhibited the highest levels of hikikomori and parental pressure.

Regarding family economic status, a significant difference was observed only in hikikomori scores (*p* < 0.01). Students reporting a high economic status had significantly lower hikikomori scores compared to those from low-income backgrounds, with a small to moderate effect size (*η*^2^ = 0.03). For mother’s educational level, significant differences were found in school climate and parental support scores (*p* < 0.05), both associated with small effect sizes (*η*^2^ values ranging approximately from 0.02 to 0.03). Finally, regarding father’s educational level, a significant difference was observed only in parental support scores (*p* < 0.01), with a small to moderate effect (*η*^2^ = 0.03).

Table 4 presents the results of the correlation analysis conducted to examine the relationships among the study variables. A significant negative correlation was found between hikikomori tendency and both school climate (*r* = −0.262, *p* < 0.01) and parental academic achievement support (*r* = −0.345, *p* < 0.01). In contrast, parental academic achievement pressure showed a significant positive correlation with hikikomori tendency *(r = 0.*237*, p < 0.*01*).*

**Table 4:** Correlation coefficients among variables.

Scale	Hikikomori	School climate	PAAP	PAAS
Hikikomori	1			
School climate	−0.262^**^	1		
PAAP	0.237^**^	−0.228^**^	1	
PAAS	−0.345^**^	0.225^**^	−0.285^**^	1

**Indicates *p* < 0.01 (two-tailed).

A hierarchical multiple regression analysis was conducted to examine whether the main predictors of hikikomori tendency remained significant after controlling for demographic background variables. In Model 1, which included the dummy-coded control variables, family economic status emerged as a significant negative predictor of hikikomori tendency (*β* = −0.132, *p* = 0.004), whereas school type was not statistically significant (*β* = 0.056, *p* = 0.227). The control variables accounted for 4.7% of the variance in hikikomori tendency. In Model 2, after entering the main study variables, school climate (*β* = −0.164, *p* = 0.001) and parental academic achievement support (*β* = −0.251, *p* < 0.001) were significant negative predictors of hikikomori tendency, whereas parental academic achievement pressure was a significant positive predictor (*β* = 0.115, *p* = 0.018). The inclusion of the main predictors resulted in a substantial increase in explained variance (ΔR^2^ = 0.142), with the full model explaining 18.9% of the variance in hikikomori tendency. Importantly, the main predictors remained statistically significant after controlling for school type and family economic status, indicating the robustness of proximal family and school factors in relation to adolescents’ social withdrawal tendencies (Table 5).

**Table 5:** Hierarchical multiple regression analysis predicting hikikomori tendency.

Predictor	B	SE	*β*	*t*	*p*	95% CI [LL, UL]
Model 1: control variables						
School Type (dummy)	0.132	0.109	0.056	1.21	0.227	[−0.08, 0.35]
Family Economic Status (dummy)	−0.335	0.117	−0.132	−2.87	0.004	[−0.57, −0.11]
Model 2: main predictors						
School Climate	−0.191	0.055	−0.164	−3.44	0.001	[−0.30, −0.08]
PAAP	0.075	0.032	0.115	2.38	0.018	[0.01, 0.14]
PAAS	−0.150	0.029	−0.251	−5.14	< 0.001	[−0.21, −0.09]

Model 1 includes dummy-coded control variables (school type and family economic status). Model 2 includes the main predictors. Model 1: *F*(4, 398) = 4.901, *p* = 0.001, R^2^ = 0.047. Model 2: *F*(7, 395) = 13.091, *p* < 0.001, R^2^ = 0.189, ΔR^2^ = 0.142.

## 4 Discussion

The findings of the study reveal significant relationships between individuals’ perceived academic success, educational background, and family economic status and their levels of hikikomori tendency. From an academic perspective, individuals who perceived themselves as academically successful had significantly lower hikikomori scores compared to those who were uncertain about their academic performance or perceived themselves as unsuccessful. This finding suggests that a strong sense of academic competence is associated with greater social engagement, and that a positive perception of academic success is linked to lower levels of social withdrawal. Similarly, individuals who had repeated a grade demonstrated significantly higher hikikomori scores, indicating that experiences of academic failure were associated with less favorable psychosocial adjustment patterns. A competitive academic system, combined with high familial expectations, may undermine students’ perceptions of their academic competence and thereby increase their tendency toward social withdrawal (Furlong, 2008; Kato, 2020). In particular, students who experience failure during their educational journey may withdraw from academic and social environments due to anxiety related to not meeting societal norms. Importantly, hierarchical regression analyses further demonstrated that the associations between parental academic variables, school climate, and hikikomori tendency remained significant even after controlling for school type and family economic status. This finding indicates that the observed relationships are robust and not merely attributable to structural differences in students’ socioeconomic or institutional backgrounds. Notably, among the examined predictors, parental academic achievement support emerged as the strongest unique correlate of hikikomori tendency. This pattern suggests that supportive and autonomy facilitating parental practices may play a more prominent protective role in adolescents’ social engagement than the risk associated with academic pressure alone. From an ecological perspective, these findings underscore the salience of proximal relational resources within the family context in shaping adolescents’ withdrawal related tendencies.

Although the mean HQ-25 total score exceeded the recommended cutoff value, this finding should be interpreted with caution. The HQ-25 cutoff score is intended as a screening indicator rather than a diagnostic threshold, particularly in nonclinical, school attending adolescent samples. Accordingly, elevated HQ-25 scores in the present study are best understood as reflecting increased vulnerability to social withdrawal and related risk tendencies, rather than clinical hikikomori.

Importantly, the present findings should be interpreted in light of the qualitative distinction between hikikomori tendencies assessed in a school attending community sample and clinically defined hikikomori. Clinical hikikomori is characterized by severe and prolonged social withdrawal with marked functional impairment, whereas school-based samples are structurally less likely to include individuals meeting the full clinical picture; thus, the HQ-25 in this study should be considered a screening tool capturing variability in withdrawal related experiences and risk markers rather than a diagnostic instrument. A recent systematic review and meta-analysis shows that individuals with hikikomori exhibit higher internalizing, externalizing, and thought problem symptoms, as well as poorer interpersonal and communication functioning than non-hikikomori controls (Nonaka et al., 2025). Moreover, a comprehensive review of adolescents and young adults highlights heterogeneity in clinical presentations and frequent co-occurrence with psychiatric symptoms/disorders (Pupi et al., 2025). Accordingly, the present results reflect early risk markers of social withdrawal and associated family and school contextual factors rather than clinically identified hikikomori with severe and chronic impairment.

Moreover, experiences of bullying have been shown to adversely affect individuals’ academic engagement and psychosocial adjustment and are therefore considered a significant risk factor in the development of hikikomori. In this context, academic pressure especially when coupled with the rigidity of the education system can be regarded as a key determinant that directly impacts individuals’ social and psychological wellbeing. The literature includes numerous studies emphasizing the influence of academic failure on social withdrawal and suggesting that negative experiences within the educational process may contribute to the development of hikikomori (Bonnaire and Roignot, 2023; Concina et al., 2024; Furlong, 2008; Kato, 2020; Teo, 2010).

When the research findings are evaluated from an economic perspective, it is evident that family economic status is significantly associated with individuals’ levels of hikikomori tendency. Participants who identified their economic status as high had significantly lower hikikomori scores compared to those who reported a low economic status. This finding suggests that economic security is associated with higher levels of social functioning, highlighting the importance of socioeconomic resources in relation to tendencies toward social withdrawal. The literature includes several studies that have examined the influence of socioeconomic factors on the development of hikikomori (Nonaka and Sakai, 2021; Nonaka et al., 2020; Umeda and Kawakami, 2012). In addition, the present study found that individuals whose mothers were not employed had higher hikikomori scores than those whose mothers were working. This outcome reflects the potential influence of parental participation in the workforce on the individual’s psychosocial processes. Particularly, a parent’s employment status may be considered a key factor that shapes the dynamics of social connectedness and interaction with the outside world. Taken together, the findings underscore the impact of academic and economic variables on individuals’ tendencies toward social isolation, and they point to the importance of enhancing psychosocial support mechanisms within educational processes. Furthermore, the results highlight the need for interventions aimed at mitigating the effects of socioeconomic disparities on individuals’ levels of social participation (Nonaka and Sakai, 2021; Umeda and Kawakami, 2012). These findings can be interpreted from an ecological perspective, suggesting that adolescents’ social withdrawal is shaped not only by individual characteristics but also by proximal contexts such as family and school. Accordingly, hikikomori tendency may be understood as a context related pattern rather than solely an individual outcome.

### 4.1 The impact of school climate on hikikomori tendency

The findings of the study revealed a significant negative association between school climate and hikikomori tendency among high school students. This result suggests that more supportive school environments are associated with lower levels of social withdrawal among adolescents. School climate encompassing multidimensional elements such as teacher-student interaction, peer relationships, the overall psychological atmosphere, disciplinary practices, and a sense of belonging has been widely linked to students’ social and emotional development in the literature (Akyol et al., 2017; Cebrián et al., 2024; Hoy and Miskel, 2005; Loukas et al., 2006; Sebastian and Allensworth, 2012).

Conversely, inadequate teacher support, peer bullying, excessive academic pressure, and exclusionary school structures have been associated with weaker school belonging and higher levels of social withdrawal. In this regard, Kato (2020) emphasized the strong association between school experiences and the emergence of hikikomori, noting that negative school related experiences are frequently observed alongside intensified social withdrawal. Supporting this view, Concina et al. (2024) reported that teachers perceive school climate as an important contextual factor related to students’ social withdrawal, highlighting the relevance of supportive relationships.

Similarly, Sugai (2016) found that school-based stressors such as intense performance pressure, conformity demands, and bullying are associated with elevated risks of social isolation, particularly in the Japanese educational context. Pozza et al. (2019) also noted that adverse school experiences are commonly observed in conjunction with hikikomori related withdrawal patterns. In the South Korean context, Lee et al. (2015) demonstrated that academic pressure, bullying experiences, and insufficient social support were jointly associated with higher levels of social withdrawal among adolescents. Taken together, these findings suggest that a safe, inclusive, and psychologically supportive school climate is closely related to adolescents’ sense of belonging and social functioning, underscoring its relevance for preventive efforts addressing hikikomori tendencies.

### 4.2 The impact of parental academic achievement support on hikikomori tendency

The findings of the study indicate a significant negative association between perceived parental academic achievement support and hikikomori tendency among high school students. This result suggests that higher levels of perceived family support are associated with better social functioning in adolescents. Consistent with this pattern, lower levels of parental support have been linked in the literature to feelings of insecurity in social interactions, decreased motivation, and higher levels of social withdrawal (Demirel and Yücel, 2017; Nonaka et al., 2022). Supportive family relationships have been associated with stronger social bonds, whereas lower levels of emotional support have been related to greater social isolation (Alyüz, 2020; Ergüden and Çelik, 2020; Yoldaş and Demircioğlu, 2019). Nonaka et al. (2020) similarly emphasized that functional parent child interactions are associated with higher levels of social engagement, whereas disconnected family relationships tend to co-occur with social withdrawal.

In line with these findings, Hamasaki et al. (2022a) highlighted associations between communication difficulties and the severity of hikikomori, while Kato et al. (2011) noted that positive parental involvement is commonly observed alongside lower levels of social isolation, loneliness, and depressive symptoms. The present findings further suggest that not only emotional support but also academic support from parents is meaningfully related to adolescents’ social adjustment. Previous research has shown that parental behavioral and cognitive involvement in the educational process is associated with higher levels of self-regulation (Grolnick and Slowiaczek, 1994), resilience, and learning motivation, particularly when such support is delivered in autonomy supportive ways (Moroni et al., 2015; Hill and Tyson, 2009).

In this context, the content and delivery style of parental support appear to be particularly relevant. Fan and Williams (2010) reported that academically supportive parental behaviors are associated with higher self-efficacy and classroom engagement among students. However, Cheung and Pomerantz (2011) cautioned that controlling forms of support characterized by excessive expectations are associated with lower intrinsic motivation and poorer psychosocial adjustment.

### 4.3 The impact of parental academic achievement pressure on hikikomori tendency

The findings of this study indicate a significant positive association between parental academic achievement pressure and hikikomori tendency among high school students. This pattern suggests that higher levels of controlling parental attitudes are associated with greater tendencies toward social withdrawal in adolescents. In particular, authoritarian, intrusive, and overly controlling parenting styles have been linked to lower levels of autonomous decision-making and weaker social engagement (Funakoshi and Miyamoto, 2015). Similarly, Nonaka et al. (2020) emphasized that parental communication styles characterized by high control are frequently observed alongside difficulties in social adjustment, especially in individuals from dysfunctional family environments.

A growing body of research has documented consistent associations between coercive parental attitudes and hikikomori related behaviors. Studies by Teo et al. (2015), Koyama et al. (2010), and Suwa and Suzuki (2003) have reported that heightened parental pressure commonly co-occurs with more severe social withdrawal patterns. Hamasaki et al. (2022b) further noted that parental pressure may manifest in forms such as overprotectiveness or emotional distance, both of which are associated with increased levels of isolation. These findings suggest that parental academic achievement pressure is often embedded in broader relational dynamics involving psychological control, emotional unavailability, and limited autonomy support.

In particular, pressure centered on academic success has been associated with adolescents’ tendencies to base self-worth on conditional approval and performance related standards (Assor et al., 2004; Roth et al., 2009). Pomerantz and Wang (2009) reported that such pressure is linked to lower levels of perceived autonomy, while Soenens and Vansteenkiste (2010) found that adolescents exposed to conditional parental regard are more likely to report feelings of guilt, inadequacy, and diminished self-worth. Schiffrin et al. (2014) similarly observed that higher academic pressure is associated with greater withdrawal from social relationships, particularly among university students.

Taken together, the present findings suggest that excessive parental academic achievement pressure represents a salient environmental correlate of adolescents’ social withdrawal tendencies. Rather than implying causality, these results highlight consistent relational patterns whereby performance-based parental expectations are linked to reduced intrinsic motivation, lower psychological flexibility, and diminished social engagement. Such patterns may help explain why adolescents exposed to high levels of academic pressure are more likely to report hikikomori related tendencies within school-attending populations.

## 5 Conclusion

The present study utilized a multidimensional approach to examine the relationships between key social environmental variables and hikikomori tendency among high school students. The specific social environmental variables included in the study were parental academic achievement pressure, parental academic achievement support, and school climate. The findings suggest that perceived parental support and a positive school climate are associated with lower levels of students’ social withdrawal behaviors, whereas academic achievement pressure from parents is associated with higher hikikomori tendencies. The results of the multiple regression analysis showed that these three variables together explain a significant portion of the variance in hikikomori tendency (*R*^2^ = 0.17).

According to the results, parental support appears to be associated with individuals’ feelings of security in their social relationships, while excessive academic pressure from parents may be linked to weaker perceptions of autonomy and higher tendencies toward withdrawal from social interactions. Similarly, a positive perception of school climate may be associated with stronger feelings of school belonging and psychological resilience, thereby relating to lower levels of social isolation.

These findings indicate that family and school-based environmental factors play an important role in adolescents’ social connectedness, psychological flexibility, and social functioning. However, given the cross sectional design of the study, these relationships should be interpreted as correlational rather than causal. From an ecological perspective, these results suggest that adolescents’ social withdrawal is shaped by the interaction of family and school environments rather than by individual factors alone. In this sense, hikikomori tendency can be understood as a context related outcome emerging from ongoing environmental conditions. In conclusion, educational environments in which academic pressure is alleviated, supportive parenting practices are encouraged, and inclusive school climates are cultivated may be relevant for promoting adolescents’ psychosocial adjustment and social participation. By addressing hikikomori tendency in a comprehensive manner within the Turkish context, this study provides an empirical basis for developing preventive strategies for families, educators, and policymakers.

## 6 Limitations

Although this study provides important findings regarding the environmental factors that predict hikikomori tendency among high school students, several limitations should be acknowledged. The research employed a cross-sectional design, which restricts the ability to determine causal relationships between variables. Accordingly, the findings should be interpreted at the correlational level and do not permit firm conclusions about cause and effect. All data were collected through student self-reports, which may introduce social desirability bias and measurement error associated with individual differences in perception. The study was conducted in the province of Istanbul and included students from selected types of high schools; therefore, the generalizability of the findings to the broader population of Türkiye may be limited. Because the data were obtained at a single time point, the temporal ordering of the variables could not be established, further constraining causal interpretation. In addition, the findings should be considered within the Turkish cultural context, where academic success, family expectations, and school related competition occupy a particularly salient role in adolescents’ educational experiences.

Although school type and family economic status were included as control variables in the hierarchical regression model, other potentially relevant covariates (e.g., gender, grade level, self-perceived academic success, grade repetition, and absenteeism) were not entered into the model. Therefore, residual confounding cannot be fully ruled out, and the findings should be interpreted with this consideration in mind. Finally, hikikomori tendency in this study was assessed based on behavioral inclinations rather than clinical diagnostic criteria. The results should therefore be interpreted as reflecting risk tendencies associated with social withdrawal rather than evidence of a psychiatric syndrome.

## 7 Implications and suggestions for future research

This study examined several social environmental variables that are associated with hikikomori tendency among high school students. However, due to the cross sectional nature of the research, it was not possible to establish causal relationships between the variables. Therefore, future studies should consider using longitudinal research designs to track the development of social withdrawal behaviors over time.

Because all variables were measured at a single time point, the temporal ordering between family and school related factors and hikikomori tendency could not be established, which limits causal interpretation. In addition, future research should take cultural context into account when examining hikikomori tendency. In Türkiye, academic success is often closely tied to family expectations, social status, and high stakes examination systems. These cultural characteristics may shape how parental academic pressure, parental support, and school climate are perceived and experienced by adolescents. Accordingly, future studies would benefit from theoretical frameworks that integrate cultural norms, family dynamics, and school related stress when explaining social withdrawal tendencies.

Additionally, qualitative research methods may provide deeper insights into students’ subjective experiences with hikikomori. Integrating these with quantitative findings could yield more comprehensive and nuanced results. Future studies may also incorporate individual level variables, such as loneliness, social anxiety, and self-esteem, as well as patterns of digital interaction, to better explain social withdrawal behavior. Conducting research with larger and more diverse samples from different regions and school types would enhance the generalizability of findings. In terms of practical implications, there is a clear need for comprehensive, school-based intervention models that support students’ psychosocial wellbeing. In Türkiye, where school social work services are not yet institutionalized, such services could play a critical role in the early identification and appropriate referral of students at risk for social withdrawal. In this regard, integrating school social work into the national education system through a formal legal framework is of particular importance.

Moreover, school guidance services should be supported not only through individual interventions, but also via classroom-based preventive programs. Teachers should receive in service training to help them recognize signs of social isolation. Mental health professionals should develop models that serve as a bridge between schools and families and conduct awareness raising and educational programs targeting parents. Overall, the development of coordinated support mechanisms that involve both families and schools, and that prioritize students’ social development, may prove effective in preventing social withdrawal behaviors such as hikikomori.

## Data Availability

The raw data supporting the conclusions of this article will be made available by the authors, without undue reservation.
